# Toward the Use of Temporary Tattoo Electrodes for Impedancemetric Respiration Monitoring and Other Electrophysiological Recordings on Skin

**DOI:** 10.3390/s21041197

**Published:** 2021-02-08

**Authors:** Silvia Taccola, Aliria Poliziani, Daniele Santonocito, Alessio Mondini, Christian Denk, Alessandro Noriaki Ide, Markus Oberparleiter, Francesco Greco, Virgilio Mattoli

**Affiliations:** 1 Center for Micro-BioRobotics, Istituto Italiano di Tecnologia, Viale Rinaldo Piaggio 34, Pontedera, 56025 Pisa, Italy; aliria.poliziani@santannapisa.it (A.P.); alessio.mondini@iit.it (A.M.); 2 Future Manufacturing Processes Research Group, School of Mechanical Engineering, Faculty of Engineering, University of Leeds, Leeds LS2 9JT, UK; 3 The BioRobotics Institute, Scuola Superiore Sant’Anna, Viale Rinaldo Piaggio 34, Pontedera, 56025 Pisa, Italy; 4 Department of Excellence in Robotics & AI, Scuola Superiore Sant’Anna, Piazza Martiri della Libertà, 33, 56127 Pisa, Italy; 5 Emerging Application Department, MED-EL Elektromedizinische Geräte Gesellschaft m.b.H., Fürstenweg 77a, 6020 Innsbruck, Austria; Daniele.Santonocito@medel.com (D.S.); Christian.denk@medel.com (C.D.); Noriaki.ide@medel.com (A.N.I.); Markus.Oberparleiter@medel.com (M.O.); 6 Institute of Solid State Physics, NAWI Graz, Graz University of Technology, Petersgasse 16, 8010 Graz, Austria

**Keywords:** soft electronics, conformable electronics, tattoo electronics, wearable sensors, PEDOT:PSS

## Abstract

The development of dry, ultra-conformable and unperceivable temporary tattoo electrodes (TTEs), based on the ink-jet printing of poly(3,4-ethylenedioxythiophene) polystyrene sulfonate (PEDOT:PSS) on top of commercially available temporary tattoo paper, has gained increasing attention as a new and promising technology for electrophysiological recordings on skin. In this work, we present a TTEs epidermal sensor for real time monitoring of respiration through transthoracic impedance measurements, exploiting a new design, based on the application of soft screen printed Ag ink and magnetic interlink, that guarantees a repositionable, long-term stable and robust interconnection of TTEs with external “docking” devices. The efficiency of the TTE and the proposed interconnection strategy under stretching (up to 10%) and over time (up to 96 h) has been verified on a dedicated experimental setup and on humans, fulfilling the proposed specific application of transthoracic impedance measurements. The proposed approach makes this technology suitable for large-scale production and suitable not only for the specific use case presented, but also for real time monitoring of different bio-electric signals, as demonstrated through specific proof of concept demonstrators.

## 1. Introduction

The terms “epidermal electronics”, and “electronic tattoos” refer to a class of electronic devices with physical properties (i.e., elastic modulus, thickness, bending stiffness and areal mass density) that approximate those of the epidermis, thereby enabling a non-invasive but intimate coupling with the complex features of the skin [1,2]. Electronic devices conformally mounted on the skin can provide a versatile and efficient means to acquire information about the body through the monitoring of biologically relevant chemical and physical variables. This can enable applications for continuous health monitoring, as well as for robotic feedback and control, prosthetics and rehabilitation, and human/computer interfaces [3,4,5,6,7,8].

Superior adhesion of devices to the skin is achieved by making substrates soft and thin. One of the most promising approaches for epidermal electronics is the development of multilayer organic thin-film structures, assembled on ultrathin polymeric membranes [3,9,10,11]. These structures are subsequently laminated onto the epidermis, conformally adhering to it. Recently, commercially available temporary tattoo paper has attracted the attention of many researchers as a platform for device integration [12,13,14,15,16,17,18,19,20,21]. Epidermal electronics fabricated on top of decal transfer paper enable an easy manipulation and reliable release/transfer on the skin (or other target surfaces), which are crucial from an end-user standpoint. In particular, temporary tattoo electrodes (TTEs) technology has been successfully demonstrated to work as a promising technology for surface electrophysiological (sEP) recordings, including electromyography (EMG), electrocardiography (ECG) and electroencephalography (EEG), overcoming the limitations of traditional disposable Ag/AgCl electrodes [13,14,18,19,22]. These ultrathin nanosheets are composed of conducting polymer complex poly(3,4-ethylenedioxythiophene) polystyrene sulfonate (PEDOT:PSS) printed on top of a thin (~500 nm) ethylcellulose layer, releasable from a decal transfer paper.

Despite the tremendous progress, the emergent field of epidermal electronics is still facing many challenges toward adoption in real world scenarios. The interfacing of stretchable and skin-conformal devices with hard electronic components, such as interconnects, memory, energy devices and wireless electronics for data collection and communication, is of major interest and has attracted intensive worldwide research efforts [23,24]. Several materials and interface design strategies, such as buckled [25,26], serpentine-shaped [27], and micro-patterned [28] design, have been reported in the literature to improve soft/hard material interfaces, preventing failure of electromechanical properties under mechanical deformation and achieving reliable device performance. As concerns the dry-electrode tattoo-like technology, the integration of easy-to-handle suitable interconnections with external devices remains, somehow, an open point that needs to be addressed, toward application in long term (e.g., longer than few hours) real-time electrophysiological recordings. The present work demonstrates the use of TTEs in a specific relevant real-word application, the monitoring of respiration through transthoracic bio impedance recording. A new approach to suitable TTE connections with external electronics devices is proposed, based on the synergic application of soft screen-printed Ag ink and magnetic interlink. This design guarantees a stable, easy to handle interconnection mechanism that enables multiple connections/disconnections without impairment of sensors integrity and functionality. The system has been further tested with additional body signal recording proof-of-concept demonstrators (EMG, ECG).

## 2. Case study, Ultrathin Films and Interconnection Technology

The respiratory rate (f_R_) is a very informative vital sign responding to a variety of stressors. In clinics, f_R_ is a typically monitored health parameter, especially in the case of chronic respiratory diseases; it is also a predictor of potentially serious adverse events (e.g., cardiac arrest, sleep apnea, and infant death syndrome) [29]. Moreover, the monitoring of f_R_ in occupational settings or sporting activities can provide useful information to improve health, well-being, and safety. Indeed, f_R_ is sensitive to physical effort, cognitive load, emotional stress, environmental challenges, pain, and discomfort, among other factors [30]. Continuous respiration monitoring can be achieved through different methods [30,31], which include techniques based on chest wall movement analysis such as strain sensors and bio-impedance sensors [32,33]. Within this framework, the revolution of stretchable electronics and sensors has recently brought significant advancements toward continuous monitoring of respiration signals in the era of real time and portable healthcare systems. A comprehensive review about wearable stretchable physical sensors for this purpose has been recently published [34].

**Figure 1:**
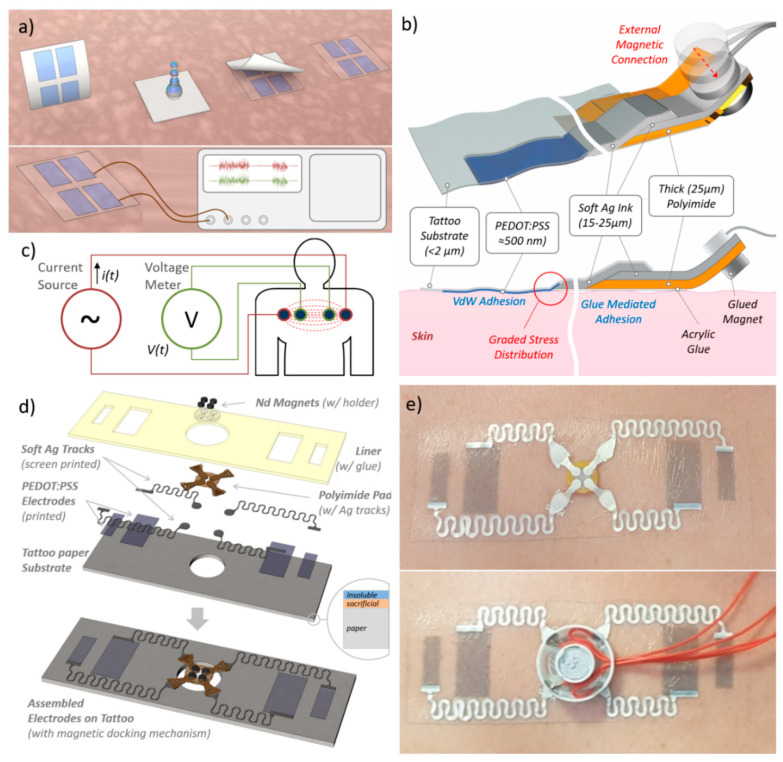
(**a**) Schematic representation of transfer on skin of printed temporary tattoo electrodes (up) and wiring to external electronic device (down). Establishing easy-to-handle/reliable and stable interconnections represents a typical challenge for any ultrathin epidermal device. (**b**) Connection schematic section for a single electrode: the use of the soft and stretchable Ag ink allows the interface between materials of different thickness and Young’s moduli, i.e., the tattoo electrode (conformally attached to the skin) and the polyimide film. The external magnetic connection allows the communication with the external device. (**c**) The specific case-study: tattoo electrodes as real time respiration sensor based on four-point measurement of bio-impedance. (**d**) Schematic representation of the design and geometry of PEDOT:PSS electrodes and interconnections for transthoracic impedance measurements. (**e**) A TTE released on the chest of a subject (top) and the same docked through a magnetic connector used for signal acquisition.

Among the different measurement strategies, bio-impedance is a non-invasive and comfortable technique for monitoring respiratory parameters when measured on the thorax. The monitoring of f_R_ using bio-impedance sensors is based on the cyclic impedance changes caused by the respiratory-induced chest wall movements and respiratory volume. During inspiration, the increase in the gas volume causes an increment of the electrical impedance of the lungs, while the expansion of the chest increases the length of the conductance paths [35]. It has been demonstrated that the relationship between this impedance change and the respiratory volume is approximately linear [30,35]. Bio-impedance sensors require skin-contact electrodes for signal acquisition from the body, positioned at the level of the upper thorax. Due to the aforementioned limitations of traditional Ag/AgCl electrodes, the continuous and long term (i.e., over more consecutive days or even for a few hours) monitoring of respiratory rate is still an open and challenging field where optimal solutions are missing. For these reasons, we propose a wearable tattoo sensor for real time respiration monitoring through transthoracic impedance measurement, both as a specific use case of clinical relevance and for validation of the proposed approach. A stable interconnection between TTEs and external rigid electronic devices (see Figure 1a) is, therefore, required.

Stable and conformal adhesion of inkjet-printed PEDOT:PSS TTEs onto skin on several locations on the body has already been demonstrated in previous works, also at the microscopic level [14]. In this work, we used a similar approach, but with different materials and assembly. In particular, we used a commercial decal transfer paper as the substrate for TTEs’ fabrication, with improved mechanical performances compared to the one used elsewhere [14]. A detailed description of this and other decal transfer paper compositions is reported in [12] and in the Materials and Methods section (see also Supporting Information SI.1). The thickness of the supporting tattoo layer, which is released on the skin after the dissolution of the water-soluble sacrificial layer and the removal of the supporting paper, was measured to be ~1.7 µm. In order to establish the external electrical connection to the TTE sensor, a 25 μm thick polyimide sheet was employed. Therefore, a mechanical mismatch between materials occurred, and caused the interface between the two materials to be fragile. Moreover, any conductive tracks either deposited or printed across the mentioned interface will result in a breakage whenever the interface undergoes a certain mechanical stress (e.g., bending or stretching), causing failure in electrical connection.

The failure at the interface between an ultrathin layer (conformally attached to the skin) and a thicker stiffer layer connected through thin conductive tracks can be caused by two different factors: flexural rigidity mismatch and the elastic modulus (Young’s modulus) mismatch. The Flexural Rigidity, *D*, is defined as the bending moment (force couple) required to bend a structure per unit length per unit of curvature. It can be defined as the resistance offered by a plate structure while undergoing bending [36]:

(1) $$1\frac{Eh^{3}}{12\left(1-v^{2}\right)}=D$$

(2) $$D\frac{d^{2}w}{dx^{2}}=-M$$

(3) D= EI

where *E* is the Young’s modulus of the material, *h* is the thickness of the beam, *ν* is the Poisson’s ratio, *M* is the internal bending moment of the beam, *d*^2^*w*/*dx*^2^ is the local curvature and *I* is the area moment of inertia (also called second area moment) of the beam cross-section.

If we consider (as a typical example) a structure composed of two different layers characterized by different flexural rigidities and conformally attached to a curved surface, then a high concentration of the stress is generated at the interface between the two layers. This happens because of the different forces generated by the two parts in response to the same curvature. If the stress overcomes the maximum stress (in the thinner layer), breakages occur. Moreover, in a structure composed of two different layers with different Young’s moduli, high stress is generated at the interface between the two layers when a strain is applied. Consequently, cracks and breakages occur. Analogously, in more complex systems (e.g., multi-layers), high stress is generated at the different interfaces.

In order to address this issue, we followed a “graded material transition” approach, using an intermediate interconnection layer made up of a soft and stretchable silver conductor paste (commercial formulation of an elastomer and Ag nanoparticles, in the following referred as Ag ink). The approach is schematically depicted in Figure 1b. Interconnecting lines and pads, which join the PEDOT:PSS ultrathin electrodes with the thick external connectors, were deposited by screen printing of the Ag ink, both on the tattoo paper substrate (containing the PEDOT:PSS electrode) and on the polyimide film (for implementing external contacts/connections). Afterwards, the two parts (the tattoo and polyimide part) were assembled by placing a drop of the same ink between the tracks on the two parts (see Figure 1b for connection schematics). The adoption of the soft Ag ink provided a compliant interface between layers with different thicknesses and mechanical properties, thus reducing the mismatch between the different layers both in terms of flexural rigidity and Young’s moduli. Estimation of mechanical and geometrical parameters of the different layers is reported in Table SI.1 (see Supporting Information), resulting from (1) in a flexural rigidity *D* of 0.0223 × 10^−9^ N·m, 56.3 × 10^−9^ N·m and 4650 × 10^−9^ N·m, respectively, for the PEDOT:PSS, Ag and polyimide layers.

Finally, the connection of the tattoo sensor with external devices is achieved through a magnetically-enabled contact, in analogy to what presented by Rogers’s group in [37]. In our approach, small magnets, glued on the backside (polyimide side) of the Ag contact pads, squeeze the latter against conductive magnets on the external device, thus ensuring robust mechanical docking and reliable electrical contacts (see Figure 1b). In order to keep the thicker part of the tattoo (polyimide and Ag ink tracks) on the skin, an additional thin bi-adhesive acrylic glue layer (the same as that provided in the tattoo paper kit) is used.

The overall configuration makes it possible to exploit the conformal adhesion (mediated by Van der Walls force) of the PEDOT:PSS electrodes with the skin, while retaining a robust interface with external devices. The effectiveness of this solution has been tested for a specific case-study with clinical relevance, as reported in the following.

## 3. Materials and Methods

### 3.1. Tattoo Fabrication

A commercially available temporary transfer tattoo paper kit (Silhouette Tattoo Paper, Silhouette America, Lindon, UT, USA) composed of two sheets (a decal transfer paper and a glue sheet) was employed. The surface of the decal transfer paper, used as unconventional substrate for electrodes fabrication, were gently washed with a DI water jet and then dried using a compressed-air gun. In order to avoid its wetting, the back of the paper was covered with an aluminum foil and the edges protected by impermeable adhesive tape. The tattoo paper outline and central hole were cut by using a CO_2_ laser cutter (model VLS3.50 by Universal Laser System). PEDOT:PSS electrodes were deposited by inkjet printing of a solution of PEDOT:PSS aqueous dispersion (Clevios PJet 700 by Heraeus, Hanau, Germany) and glycerol (10% vol). PEDOT:PSS ink was used after filtration (Minisart, average pore size 0.20 μm, Sartorius, Göttingen, Germany). Inkjet printing was carried out with a Dimatix DMP-2800 system (Fujifilm Corp., Tokio, Japan) endowed with a 10 pL cartridge (DMC-11610). Interconnection Ag tracks on tattoo paper were deposited by screen printing of a stretchable Ag conductor paste (CI-1036, Engineered Materials Systems). For the screen printing, we used a homemade setup for manual screen printing, composed of a mask (laser cut stencil) and a blade for squeegeeing and uniformly distribute the paste. After the deposition, a baking at 120°C for 10 min was performed. A 25 μm thick polyimide foil (Kapton HN, Wilmington, DE, USA, acquired from RS) was employed as support layer for the external electrical connection (contact pad). The outline of the contact pad was cut using a CO_2_ laser cutter (model VLS3.50 by Universal Laser System). Silver pads on contact pad were deposited by screen printing of the stretchable Ag conductor paste through a stencil mask. After the deposition, a baking at 120 °C for 10 min was performed. Contact pads were flipped for correct assembly. During the assembly of the two parts, the Contact pad was glued to the tattoo putting a small drop of the Ag paste between the tracks and the pads, and then baked at 120 °C for 10 min. The glue sheet of the temporary transfer tattoo paper kit was firstly laser cut, and then placed on top of the device for providing additional tattoo substrate adhesion (except for the sensing PEDOT:PSS parts) while acting as protective insulating layer, preventing direct contact of interconnection lines with skin. Four small neodymium magnets (0.5 mm thick, 2.5 mm diameter) were fixed with glue on a plastic support (0.5 mm thick) to obtain a magnetic disk. The magnetic disk was finally attached to the on-tattoo mounted contact pad, on the opposite side of the silver pad, thus obtaining the final sensor assembly which was ready for use.

### 3.2. Tattoo Characterization

Surface analysis was performed on tattoo samples, recollected onto different specific supports, after the release in water as free standing membrane. Thickness measurements were carried out with a P6 stylus profilometer, KLA Tencor, onto samples recollected on clean a Si wafer, following the procedure described in [38].

### 3.3. In-Lab Stretching Test to Evaluate the Connection Reliability

Stretching tests were performed to evaluate the connection reliability of the proposed TTE on a customized test platform, specifically built for the scope, since a quantitative evaluation of these aspects could not be directly performed on human subjects, because of poor repeatability of the movements, either by the same subject or among different subjects. The platform was implemented to simulate the chest expansion/contraction related with breathing. Since specific and standard tests do not exist for this application, a customized experimental protocol for the electromechanical testing of TTEs was implemented. The experimental protocol included three steps: (i) the TTE shall be released onto a stretchable and conductive substrate that simulates the surface of the chest wall; (ii) the substrate shall be deformed in a way that simulates the deformation of the chest during respiration; (iii) together with the above mentioned deformation, a vertical acceleration shall also be considered to simulate real-life scenarios such as breathing during walking and/or running.

**Figure 2:**
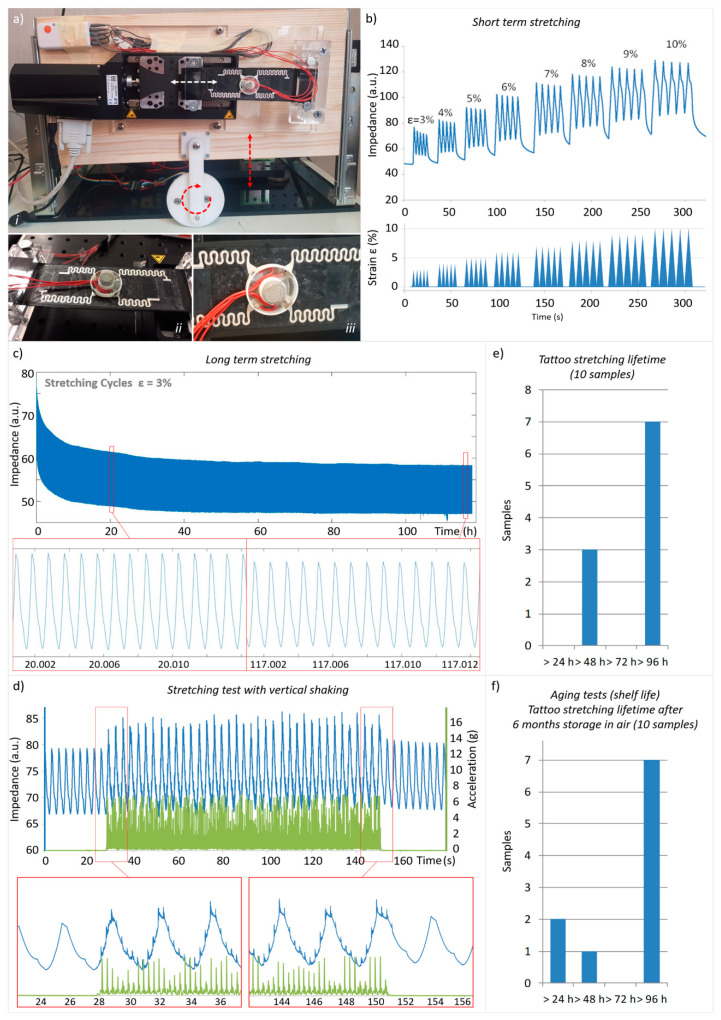
(**a**) A picture of the experimental setup used for stretching test (top) and of the TTE transferred onto a conductive and stretchable rubber substrate (bottom). (**b**) Representative example of short term stretching test. (**c**) Representative example of long term stretching test. (**d**) An example of the impedance signal during 2 min of “shaking”, showing that the sensor output is scarcely affected by motion artifacts. (**e**) Summary of the results of long term stretching test. (**f**) Summary of the results of long term stretching showing that stretching lifetime of the tattoo is not significantly deteriorated with aging.

*Platform implementation*—Conductive rubber samples (Axel 2-9330-02) were fixed at one edge and clamped to a motor driven translation stage on the other (L-509, Physik Instrumente) in order to apply a uniaxial tensile deformation along the transverse axis. The control and conditioning electronics related to the translation and testing stage were connected through a multichannel DAQ Board (model USB-6218, National Instruments, US) with a dedicated PC. Stage position control was performed using a customized Graphical User Interface (GUI) developed with Visual Studio 2010 (Microsoft, Redmond, CA, USA). The setup for the stretch was fixed on a vertical plate which can move along y axis. A gearmotor (100:1 Metal Gearmotor 37D × 73L mm with 64 CPR Encoder) was employed for vertical movement. TTE was transferred on fixed conductive rubber just before the start of experiments, by gently wetting the backside of the TTE sample placed on the rubber surface with a wetted sponged for removing the paper support. Impedance measurements were performed by using a Shimmer 3 ECG wireless sensor unit (Shimmer, Ireland), configured for 4-point impedance measurement, with excitation AC current of 30 μA @64 kHz, signal acquisition resolution < 1nV (24-bit ADC, 1.6 V full span), input referred noise < 15 μV_PP_ at all working conditions. The Shimmer unit was electrically connected with the TTE trough a wired magnetic docking pad (as shown in Figure 2a). Sampling rate was fixed as specified in the specific protocols.

*Stretching test protocols* —Two types of tests were carried out on TTE Samples: “short term” and “long term” stretching tests.

Short term tests were designed to evaluate the range of stretchability of the TTEs, applying a uniaxial tensile deformation to the TTE/substrate assembly. In detail, short-term stretching tests were performed on a set of 10 TTE samples, applying the following stretching sequence: 3–4–5–6–7–8–9–10% (every deformation repeated 5 times, stretching speed was fixed to about 2% s^−1^). Sampling rate was fixed at 300 Hz.

Long term stretching tests were designed for the evaluation of the durability: a cyclic uniaxial tensile deformation was applied to the TTE/substrate assembly up to 96 h, using a stretching amplitude and rate which simulate the typical respiratory process for a healthy adult, i.e., 3% and 18 cycles/min, respectively. Long-term stretching tests were performed on a set of 10 TTE samples.

During the long term stretching test, a vertical vibration (acceleration range 4 g) of 2 min was applied at constant time intervals of 1 h to mimic walking/running movements. The sampling rate was fixed at 1 Hz.

Long term stretching tests were repeated on a batch of 10 TTE samples stored in air at room temperature for 6 months, to evaluate the effect of aging on the samples. The sampling rate was also fixed at 1 Hz in this case.

### 3.4. Tests on Volunteer

#### 3.4.1. Transthoracic Impedance Measurement with Commercial Sensor Unit

A volunteer, chosen from among the authors, performed a series of standardised routines simulating the daily human real life activities while simultaneously monitoring the transthoracic impedance and the breathing through a thermistor placed close to the nose. The measurements were performed with a commercially available wireless device, namely the Shimmer 3 ECG wireless sensor unit (Shimmer, Ireland). The volunteer was prepared as reported in the following steps: (1) The skin of the participant is gently rubbed with an antibacterial solution to remove the natural dermal fat coating; (2) the TTE is applied in correspondence of the volunteer’s sternum, at the Louis angle; (3) a Negative Temperature Coefficient (NTC) thermistor (Alice 6 respiratory sensor nasal/oral thermistor, Ternimed UG, Germany) is placed in the patient’s nose to provide breathing reference signal; (4) the Shimmer 3 ECG wireless sensor unit (Shimmer, Ireland), configured for 4 point impedance measurement, (excitation AC current of 30 μA @64 kHz, and sampling rate of 256 Hz), is placed right up the patch by using a double layer adhesive tape; (5) the Shimmer3 is wired-connected to the thermocouple and to the TTE’s though specifically built magnetically docking cables.

The measurement protocol used in this investigation emulated the daily human activities, including six manoeuvres that represent the typical actions performed during a normal 24 hours’ real life day. In detail, the 7 minutes’ measurement protocol includes: short-time breath-holding (up to patient ability/up to max 30 s); normal breathing (60 s); loud voice reading (60 s); left/right head rotation (30 s); left/right torso rotation and inclination ahead/back (30 s); slow paced walking (2 km/h; 60 s); medium paced walking (3 km/h; 60 s); free talking (60 s). Data for transthoracic impedance and breathing were acquired by Shimmer3 during all the protocol and analysed offline. Both bio-impedance signal and thermistor signal were filtered off-line with a Butterworth zero-phase digital filter. The thermistor signal worked as a reference signal. The local maxima, and then the onsets of the respiration, were determined manually from the two filtered signals: features such as the minimum respiration cycle length and amplitude of adjacent local maxima, were used for this purpose. Finally, the detected onsets in the reference and bio-impedance signals were compared. One onset in the reference signal was considered successfully detected if the correspondent onset in the bio-impedance signal was detected within a time window of ±500 ms. The detection accuracy was defined as the number of true positive onsets that were detected with the bio-impedance, divided by the total number of true respiration onsets in the reference signal.

#### 3.4.2. EMG Measurement with Miniaturised Standalone Devices

A miniaturized standalone device was developed to investigate the possibility of recording a filtered EMG signal and of reliably detecting a fixed signal threshold. Details on the device fabrication are reported in Supporting Information and as open source in an accessible repository. For EMG detection, a specific three electrodes design was used (see Supporting Information for details). The EMG device was tested on the arm of a volunteer chosen among authors; the three-electrode tattoo was placed on the forearm in a position suitable to detect the contraction of the wrist flexor muscles, activated by the wrist flexion and hand closure. Once the EMG device was connected magnetically to the tattoo, the signal was acquired and transmitted via Bluetooth (BT) to a PC for data display and storage. Finally, qualitative assessment of the functionality and acquired signal was performed.

#### 3.4.3. ECG Measurement with Miniaturised Standalone Devices

A miniaturized standalone device was developed to investigate the possibility of recording an ECG signal from the chest. Details on the device fabrication are reported in Supporting Information and as open source in an accessible repository. For ECG detection, a specific two electrodes design was used (see Supporting Information for details). The ECG device was tested on the chest of a volunteer chosen among authors; the two-electrode tattoo was placed at the middle of the chest in correspondence with the sternum. A comparative test was then performed by using as reference a commercial handheld device (Prince 180B Easy ECG Monitor, Heal Force Bio-meditech, Shanghai, China) equipped with three standard pre-gelled electrodes: the two signal electrodes were placed just below the ECG tattoo electrodes, while the ground reference was placed on a leg. Once the ECG device was magnetically connected to the tattoo, synchronised acquisition via BT was started. The recorded signals (TTE and reference) were evaluated over a full 60 s sample in terms of root mean square (RMS) noise amplitude, as the square root of the integral of the power spectrum (at mean squared amplitude, MSA) above 40 Hz, and in terms of signal-to-noise ratio (peak-to-peak signal/RMS noise).

## 4. Results and Discussion

### 4.1. TTEs for Transthoracic Impedance Measurements Design

Transthoracic impedance measurements are performed with two or four electrodes placed on the chest of the subject. In a typical four-electrodes (tetrapolar) configuration a high-frequency and low-amplitude current (typically: 50 kHz, <1 mA) is injected by two electrodes on the thorax, whereas the other two electrodes are used to record the impedance changes by measuring the voltage changes between them [35] (see Figure 1c). Compared to the two-terminal measurement configuration, the four-electrode configuration yields a more accurate measurement because the sites of current injection and voltage measurement are physically separated [39]. In order to use TTEs for transthoracic impedance measurements, the design and the geometry of PEDOT:PSS electrodes and interconnections have been optimised for the proposed application, as illustrated in Figure 1d. Four rectangular skin-contact electrodes were realized by inkjet printing on tattoo paper of a mixture of PEDOT:PSS aqueous dispersion and glycerol. Glycerol was added as a biocompatible additive to improve the conductivity and print quality of printed films [40]. PEDOT:PSS electrodes are electrically interconnected with a more thick and resistant polyimide part (for external connection), through flexible/stretchable serpentine tracks, deposited by screen printing of the Ag ink. The use of a serpentine geometry is a good strategy to increase the stretchability of a conductive track in wearable and epidermal electronics [1]. The design of the polyimide part has been optimized in order to guarantee the connection through magnetic docking with external connectors/devices. Four small (0.5 mm thick, 2.5 mm diameter) Nd magnets (one for each pad) were fixed with glue on a plastic support to obtain a magnetic disk (Figure 1d). The magnetic disk was attached to the polyimide contact pad, on the opposite side of the Ag pads. Further details on fabrication are reported in the Materials and Methods section (see also Supplementary Materials Figure S2). Magnets on the tattoo are placed in a position that maximizes the squeezing force for planar contacts between the contact pads and the pads on the external electrical connectors. Figure 1e shows a tattoo released on the chest of a subject, and a magnetic connector used for signal acquisition. The magnetic interlocking system is able to sustain multiple connections/disconnections to the docking device without failure.

### 4.2. In Lab Stretching Test

TTEs were designed to be placed on the thorax of the subject at the sternal angle, following the respiratory-induced rhythmic movement of the chest wall. The sternal angle is an ideal spot for bio-impedance measurements, as it is characterized by a low percentage of fat in the underlying skin layer, and thinner pectoralis muscle thickness. Moreover, this is also a stable position during high body movement situations, since the bone is not part of any joint subject to roll, pitch and yaw.

Ideally the TTE should satisfy a series of requirements to be used in the proposed application. These include, among others, the possibility to be stretched over the length of the chest expansion/contraction during the respiration, without losing its functionality for at least 24 h. Additionally, the TTE, thanks to the intrinsic conformability, should prevent (or limit) the artefacts originated by the fast change of the impedance at skin-electrode interface generated by body movement. The latter can indeed induce relative displacement (sliding and local detachment) between the electrode and the skin surface, especially in the case of bulky or poorly adherent electrodes.

Since a quantitative evaluation of these aspects could not be directly performed on humans due to poor repeatability, a customized test platform has been implemented to simulate the chest expansion/contraction related with breathing. The tensile testing equipment, whose setup is described in detail in the experimental section, is shown in Figure 2a.

Conductive rubber sheets were used as substrates onto which TTEs were transferred, as shown in Figure 2a. In the deformation test, impedance through the TTEs and substrate was measured while a pre-set deformation protocol (described in Materials and Methods) was applied on the TTE/substrate assembly. Notably, the rubber substrate was selected to simulate the properties of skin: it has an elastic modulus of 9.8 MPa (vs. 4–20 MPa of skin [41]) and a variation of resistance (due to piezoresistive effect) of around 0.1–1 Ω @64 kHz for a 10% stretch (vs. 0.2 Ω to 5 Ω pk-pk, typical transthoracic impedance variation during respiration) [42], thus providing a valid test platform for the specific evaluation.

Short term tests, designed to evaluate the stretchability of the TTEs, were performed, inducing uniaxial tensile deformation up to 10% on TTE sample. This value is larger than the typical maximum skin stretching on the chest, 8.5%, due to a high inspiration act [43]. Ten TTEs were successfully tested in short term stretching conditions: no TTE did break during the stretching, and the simulated bio-impedance signal acquired by TTE did not show discontinuities or non-linearity. An example of the results obtained with a typical sample is reported in Figure 2b.

With the long term stretching tests, introduced to evaluate the duration of TTE under prolonged solicitations, we induced 3% cyclic deformations on TTE, with a frequency of 18 cycles/min (simulating the typical respiratory process) continually up to 96 h. We decided to stop the tests after 96 h even though the samples were still working, as this value greatly overestimates the minimum target value for the proposed application (24 h—TTEs are designed to be replaced daily). Figure 2c shows an example of the variation of the impedance along time. Additionally, the during long-term stretching test, a vertical acceleration (“shaking”) to mimic walking/running movements was applied at constant time intervals. The quality of the signal and the stability of interconnections during the shaking was evaluated. Figure 2d shows an example of the impedance signal during 2 min of shaking: the curve shows some signal perturbations due to the vertical movements, but the quality of the signal is still more than suitable for the specific application (see also Supplementary Video SV1 for an overview of running experimental setup). In that case, the typical Signal to Noise Ratio (SNR) of the impedance signal during the shaking phase (calculated as signal to noise standard deviations ratio) is around 4 (while whiteout shaking SNR is >50).

The results of the long-term stretching tests show the distribution of the tattoo failure time (Figure 2e). All the samples reached at least 48 h of stretching without failure, i.e., twice the minimum duration defined for the specific application (24 h). Moreover, 70% of the samples were able to achieve 96 h of stretching without failure. Failure of just three samples at 48 < t < 96 h was caused by the formation of cracks along the Ag tracks, in apparently random locations. This may be due to the presence of small defects in the tracks, caused by the manual screen printing fabrication process. The use of a professional screen printer could improve the quality of the printing, and consequently, the durability of the tracks.

The same long-term tests were also performed on aged samples, to demonstrate the stability of the TTE over time in standard storage condition. A batch of 10 TTE samples was stored in air at room temperature for 6 months. Long term test results obtained with this batch are comparable with the ones obtained with fresh samples, as reported in Figure 2f: all samples lasted more than 24 h, 70% of samples lasted more than 96 h.

### 4.3. Test on Subject

**Figure 3:**
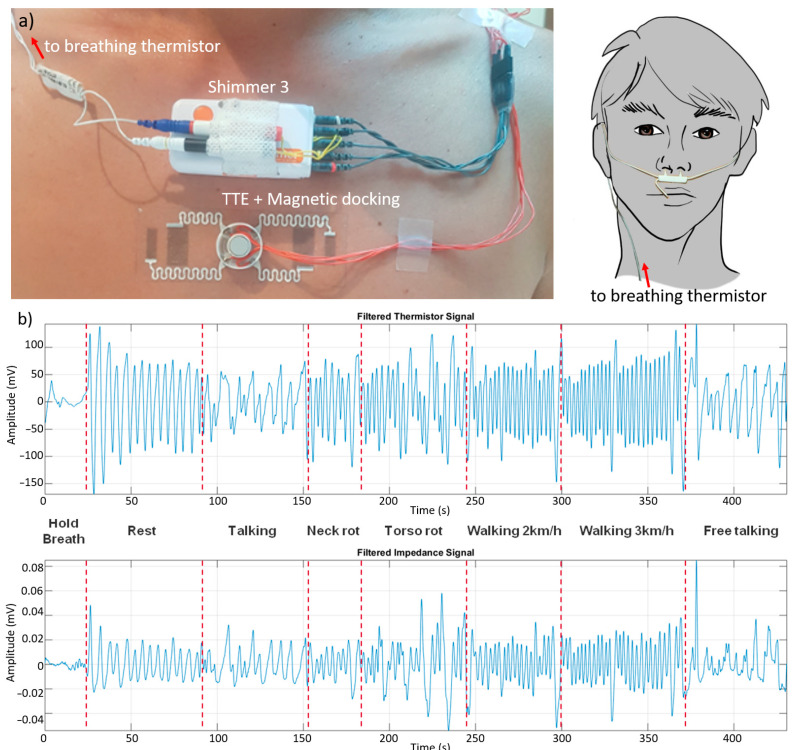
(**a**) A picture of a volunteer user wearing TTE on skin during test on humans and interconnections with Shimmer unit used for tests. A breathing thermistor was used for comparison. (**b**) Impedance variation measurements during a series of standardised routines simulating the daily human real life activities (i.e., walking) and comparison between the impedance variation acquired by TTE (top) and breathing measured through a thermistor placed close the nose (bottom).

TTEs for transthoracic impedance measurements were tested on a volunteer subject, chosen from among the authors, to evaluate the ability to acquire a reliable signal for the real-time monitoring of respiration in real life activities. The volunteer performed a series of standardised routines simulating daily activities (see detailed protocol in Materials and Methods), while acquiring the transthoracic impedance through the tattoo electrodes and, in parallel, as control, the breathing through a thermistor placed close the nose. Both devices were connected with the Shimmer unit for real time acquisition/logging of data (see Figure 3a).

Measurements are analysed off-line. Both bio-impedance signal and thermistor signal are filtered off-line to remove high frequency noise. The thermistor signal works as a reference signal, being theoretically immune to movement artefacts, even if some ripples in the signal can still occur because of complex mechanisms in the respiratory phase. Nevertheless, they are considered as not relevant for the current study. An example of the results obtained when acquiring, in parallel, impedance variation and breathing is reported in Figure 3b. The SNR of impedance signal (calculated as ration of standard deviations of signal and noise) is around 4 before filtering (>>10 after filtering), with a relative signal variation of 2–3% in rest condition. The average detection accuracy on the volunteer subject over the different manoeuvres is approximately equal to 92% (see also Supplementary Materials Figure S4 for accurate signal comparison).

The volunteer user was asked to describe whether he perceived the presence of the tattoo and if his movements were affected or hampered by the tattoo. The user claimed not to perceive the tattoo and did not describe any constraint of his natural movements. After the use, the temporary tattoo electrodes were removed by washing with soap and water and gently rubbing. The use of a commercial medical adhesive remover facilitated the removal of the tattoo.

### 4.4. Other Use-Case Examples, with Miniaturised Standalone Device

Apart from the respiration monitoring case presented so far, the proposed TTE approach is potentially suitable for a multitude of different applications, including, among others, health monitoring systems and human–machine interfaces.

**Figure 4:**
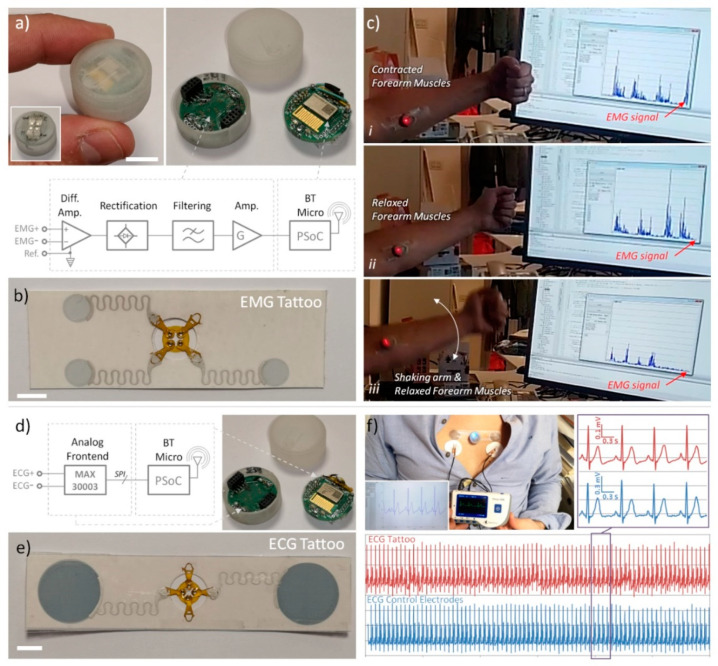
(**a**) Picture and schematic of magnetic dockable BT acquisition device, specifically designed to acquire filtered EMG signal (scale bar 1 cm). (**b**) Picture of three-electrode tattoo for EMG signal acquisition (scale bar 1 cm). (**c**) Example of use of tattoo EMG electrodes with BT device transmitting low-pass filtered data to a PC, demonstrating large immunity from motion artefacts: signal is generated exclusively by muscle contraction, not from forearm shaking. (**d**) Picture and schematic of magnetic dockable BT acquisition device, specifically designed to acquire ECG signal. (**e**) Picture of two-electrode tattoo for ECG signal acquisition (scale bar 1 cm). (**f**) Measurement of ECG carried out by using tattoo ECG electrodes with specific ECG BT device compared with commercial handhold device using three standard pre-gelled electrodes: rescaled graphs are compared in the same time framework; in the inset, the zoomed view of a small portion (3 s) of the full acquisition time (60 s) is reported. The matching is almost perfect.

To explore these possibilities, we developed a small open platform that can be magnetically docked on the TTE worn on the skin and is able to transmit the measured data over Bluetooth (BT) connection. We investigated the specific use in EMG recording with threshold detection and in ECG signal monitoring (see Figure 4). The modular device can easily adapt to different types of measurement, due to having a BT microcontroller board for data collection/elaboration and transmission, and a different sensor board implementing the analog front-end for each specific application. Details on the device fabrication, electronic schemes, firmware and software are reported in the Supporting Information and as open source in an accessible repository (see Supporting Information—Stand-alone device for Tattoo Electrode interface—Fabrication and details). Release of tattoo and operating Bluetooth module docking/connection is also shown in Supporting Video SV2.

#### 4.4.1. EMG Measurement and Threshold Detection

We investigated the possibility of recording a filtered EMG signal and of reliably detecting a fixed signal threshold. For EMG detection, we used a different tattoo design, as shown in Figure 4b. The TTE comprised three electrodes: a pair for detecting the differential signal generated by muscle contraction, the third one as reference. The analog front-end used for signal conditioning is schematically reported in Figure 4a (see Supporting Information for full details). The EMG device has been tested on the arm of a volunteer, to detect the signal generated by contraction of the wrist flexor muscles (see Figure 4c). Once the EMG BT device was connected magnetically to the tattoo, the signal was acquired and transmitted to the PC for data display and storage. When the muscles were relaxed a very low background signal was detected (in the order of few mV), while, when contracting the muscles, a very clear and high signal was reported (up to 1 V with built in hardware amplification). It is worth noting that strongly shaking the forearm without contracting the muscles resulted in a low background signal, comparable with the one produced at rest (see Figure 4c and Supporting Video SV3), thus demonstrating that the system (thanks to the magneto-electric connections and smart data acquisition) is quite robust against motion artefacts, also in “real world” conditions. The EMG BT device could find application as muscle-controlled wireless interface in several interesting scenarios, including prosthetic control [12] or interface for entertainment and gaming. As an example, muscle activated remote control of toy car was also demonstrated (see Supporting Video SV4).

#### 4.4.2. ECG Recording

We also investigated the possibility of measuring and transmitting ECG signal with a stand-alone device. A different TTE design was used for ECG detection (Figure 4e). It consisted of two bigger electrodes (2.5 cm of diameter), separated by 10 cm. Just two electrodes are sufficient, since the analog front-end chosen for the specific application has a built-in reference system (see Figure 4d and Supporting Information for full details).

The ECG device has been tested on the chest of a volunteer chosen among authors by using, as reference, a commercial handheld device with three standard pre-gelled electrodes (see Figure 4e). Once the ECG BT device was magnetically connected to the tattoo, synchronised acquisition was started. Several acquisitions, each lasting 60 s, were performed, and tattoo signal compared with reference one (see Figure 4e bottom for typical measurement results, see also Supporting Video SV5). In all the trials, the signals acquired (tattoo electrodes and gelled references) are mostly superimposable (apart for a scale factor), thus demonstrating the perfect functioning of the device. It is also worth underlining that the resolution of the ECG BT device (<1 µV) was shown to be superior compared to the commercial device resolution (10 µV), making possible the detection of smaller features of the waveform. In addition, in terms of RMS noise and signal-to-noise ratio, the Tattoo ECG BT system outperforms the reference one. In fact, estimating the RMS “noise” to be above 40 Hz [14], the amplitude was 0.29 µV with the ECG BT tattoo system and 2.00 µV with the reference device. The total RMS amplitude was, instead, 62.96 µV versus 156.70 µV, giving a signal-to-noise ratio of 211 and 78, respectively (see also Supporting Materials SI.9). Specifically, the EMG BT device could easily find application as a low cost, high accuracy personal tool for heart monitoring.

## 5. Conclusions

In this work, a new strategy for the establishment of easy-to-handle/reliable and long-term stable interconnections between ultra-conformable temporary tattoo electrodes (TTEs) and external magnetically docked devices has been presented. The proposed approach was validated on a specific real-world use case with clinical relevance, i.e., a disposable epidermal sensor for the real time monitoring of respiration. Moreover, some proof-of-concept demonstrations were presented, further demonstrating the approach for specific use in EMG recording with threshold detection and in ECG signal monitoring. The efficiency of the TTE and the proposed approach under stretching (up to 10%) and over time (up to 96 h) has been verified on a dedicated experimental setup and further demonstrated with a dedicated test on human subjects. The proposed design makes TTE technology amenable for large-scale production of low-cost skin-contact sensing devices which could be successfully exploited for a multitude of different applications, including, among others, health monitoring systems and human–machine interfaces.

## Data Availability

All data available are presented in this study and in Supplementary Materials. Row data are available on request from the corresponding authors.

## Supplementary Materials

The following are available online at https://www.mdpi.com/1424-8220/21/4/1197/s1. Supporting Information available as PDF including additional details and Figures S1–S9. All information and materials for implementing the devices, including mechanical design, electronics schematics and layout, firmware and software, are made available as open source at the following repository: https://doi.org/10.5281/zenodo.4382056 (accessed on 8 February 2021). List of SI videos: SV#1 Video of tattoo stretching test while shaking, SV#2 Tattoo electrodes release on skin with sponge and EMG signal acquisition, SV#3 EMG demo with shaking, SV#4 EMG RC car control demo, SV#5 ECG comparative demo. High resolution SI videos also available for download from here: https://gitlab.iit.it/Virgilio.Mattoli/tattoo-electrode-device/-/tree/master/SI%20Videos (accessed on 8 February 2021).
