# Supplementary material for: Toward the Use of Temporary Tattoo Electrodes for Impedancemetric Respiration Monitoring and Other Electrophysiological Recordings on Skin
Source: Sensors (Basel). 2021 Feb 8;21(4):1197. doi: 10.3390/s21041197 (PMC7915056; doi:10.3390/s21041197)
Supplement: Supplementary file 1 [file sensors-21-01197-s001.pdf]

Supporting Information

Toward the use of temporary tattoo electrodes for impedancemetric respiration monitoring and other electrophysiological recordings on skin

S. Taccola* [1,2], A. Poliziani [1,3,4], D. Santonocito [5], A. Mondini [1], C. Denk [5], A. N. Ide [5], M. Oberparleiter [5], F. Greco* [1,6] and V. Mattoli* [1]

[1]Center for Micro-BioRobotics, Istituto Italiano di Tecnologia, Viale Rinaldo Piaggio 34, 56025 Pontedera, Pisa, Italy.
[2] Future Manufacturing Processes Research Group, School of Mechanical Engineering, Faculty of Engineering, University of Leeds, Leeds LS2 9JT, United Kingdom.
[3] The BioRobotics Institute, Scuola Superiore Sant'Anna, viale Rinaldo Piaggio 34, Pontedera, 56025 Pisa, Italy
[4] Department of Excellence in Robotics & AI, Scuola Superiore Sant'Anna, Piazza Martiri della Libertà, 33, 56127 Pisa, Italy.
[5] Emerging Application Department, MED-EL Elektromedizinische Geräte Gesellschaft m.b.H., Fürstenweg 77a, 6020 Innsbruck, Austria.
[6] Institute of Solid State Physics, NAWI Graz, Graz University of Technology, Petersgasse 16, 8010 Graz, Austria.
* Correspondence: S.Taccola@leeds.ac.uk; francesco.greco@tugraz.at; virgilio.mattoli@iit.it

Details on preparation of decal transfer Silhouette paper

The composition of decal transfer Silhouette paper is (according to available datasheet): paper sheet (contents 83%); polyvinyl alcohol resin (10%); polyamide resin (3%), polyalyl resin (2%) and polyurethane resin (2%).

While the exact composition and arrangement of the different layers is not disclosed in the datasheet, our understanding and investigation make us conclude that Silhouette paper is made up of at least three layers: the paper carrier; a PVA sacrificial water soluble layer (which allows the release of the tattoo), and the tattoo layer which can be transferred onto skin.

Before electrodes and tracks deposition, the surface of the decal transfer paper has been gently washed with a water jet, and then dried using a compressed-air gun. Comparing the morphology of the paper observed by optical microscope before and after the wash, it seems that a water soluble layer on top was removed by washing (Figure SI1).

This hypothesis was confirmed by thickness measurements of the released tattoo layer without or with wash: washing of the tattoo paper sheet prior to release reduced the final thickness of the tattoo layer from about 4-4.5 μm to about 1.7 um.

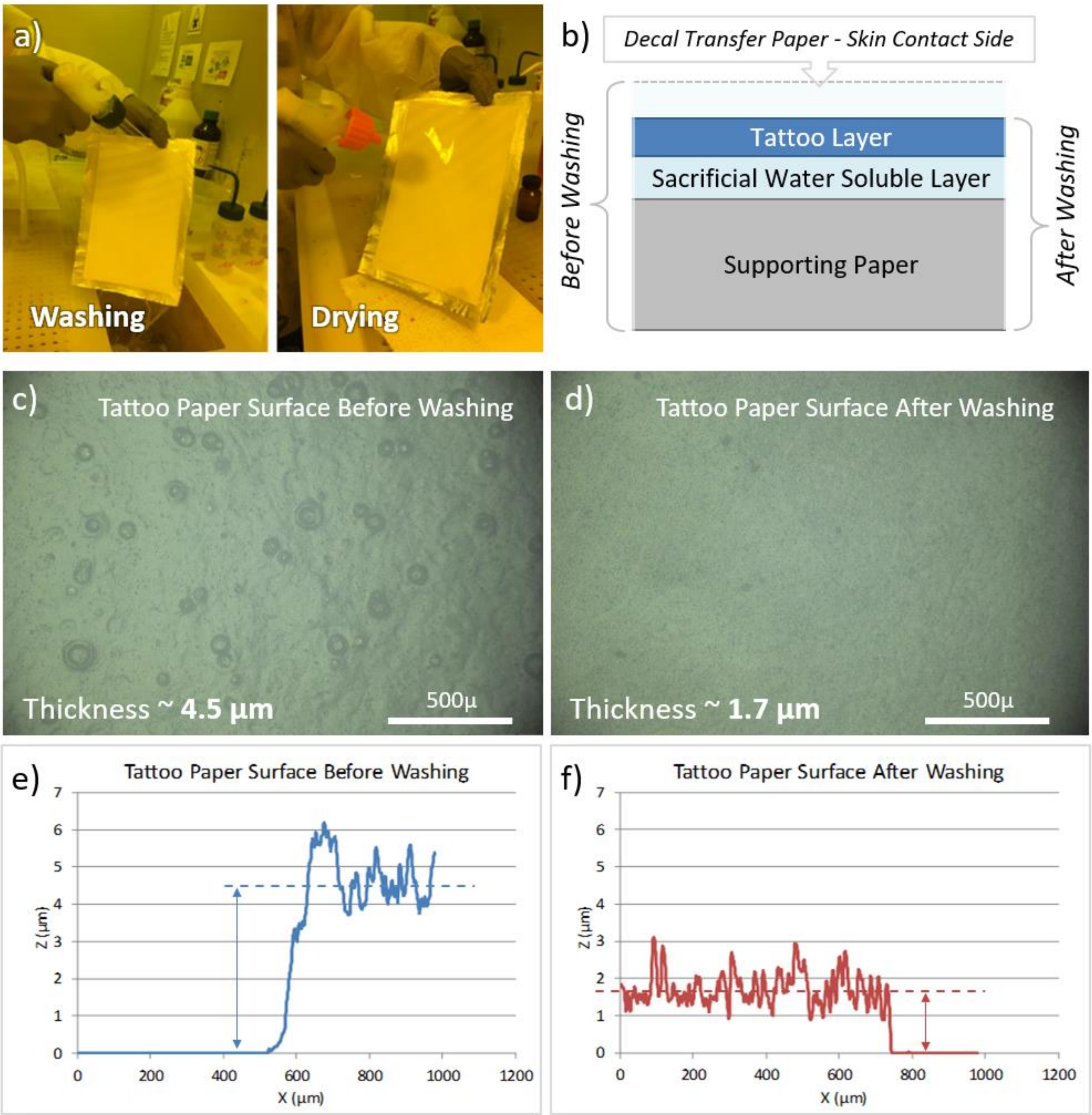

Figure 1. a) Washing and drying of the surface of the decal transfer paper using a DI water jet (left) and a compressed-air gun (right); b) A schematic representation of the different layers which compose the paper before and after washing and the corresponding optical microscope images of the paper surface before (c) and after (d) the treatment; Typical mean value for decal transfer paper thickness before and after washing are reported, together with the corresponding examples of thickness profiles (e and f).

Tattoo electrodes -fabrication procedure

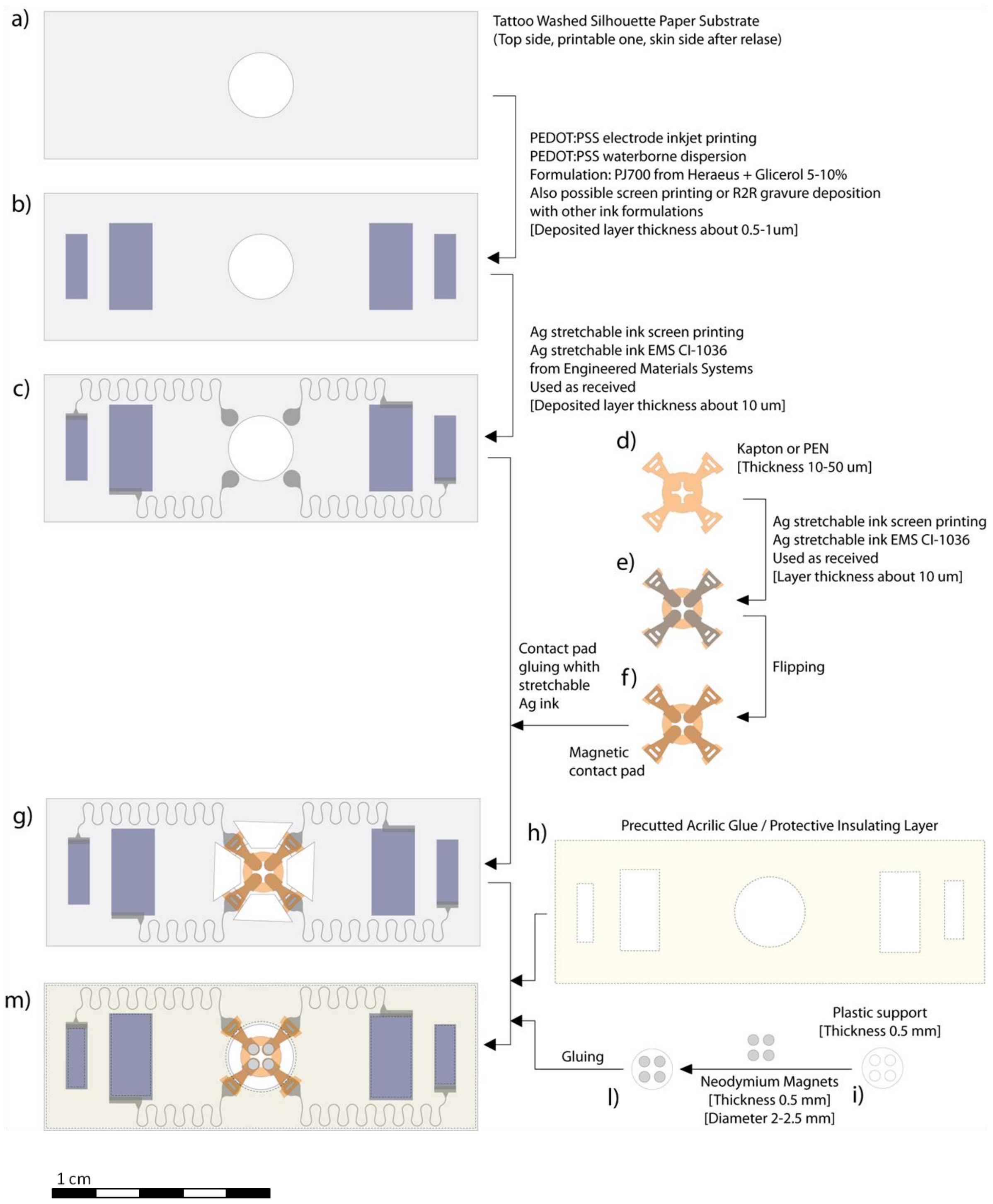

Figure 2. Schematic representation of Tattoo electrodes fabrication procedure: a) tattoo paper substrate; b) printing of PEDOT:PSS electrodes; c) Ag ink interconnection track screen printing; d) polyimide layer for supporting the external electrical connection, shaped by laser cutter; e) Ag ink interconnection track screen printing on polyimide layer; f) contact pad flipping for correct assembly; g) assembly of contact pad on tattoo electrode layer by small drops of the Ag ink; h) laser cut glue sheet (form transfer tattoo paper kit) used as passivation layer; i-l) assembly of neodymium magnets and support; m) final tattoo electrode assembly.

Tattoo electrodes - Inkjet printing and conductivity

In general, concerning ink-jet printing, the properties of the substrate strongly influence the quality of the print, and consequently the conductivity of the printed electrodes. For this reason the printing parameters have to be optimized. In particular we worked on the formulation of PEDOT:PSS ink (conducting polymer in water) using glycerol as a biocompatible additive improving conductivity and print quality (*S.H. Eom et al. Organic Electronics 10, 536–542, 2009*). The final formulation chosen was PEDOT:PSS Clevios P Jet 700 (H.C. Starck) + 10% vol glycerol. In order to print more layers, an intermediate heating at T=120°C for 10 min is needed. In Figure SI.3 it is shown how the addition of glycerol improved the quality of the print and increased the conductivity of PEDOT:PSS electrodes, printed as multiple superimposed layers. "R" is the surface resistivity in Ω/□, measured the opposite edges of a printed square of 1cm lateral size.

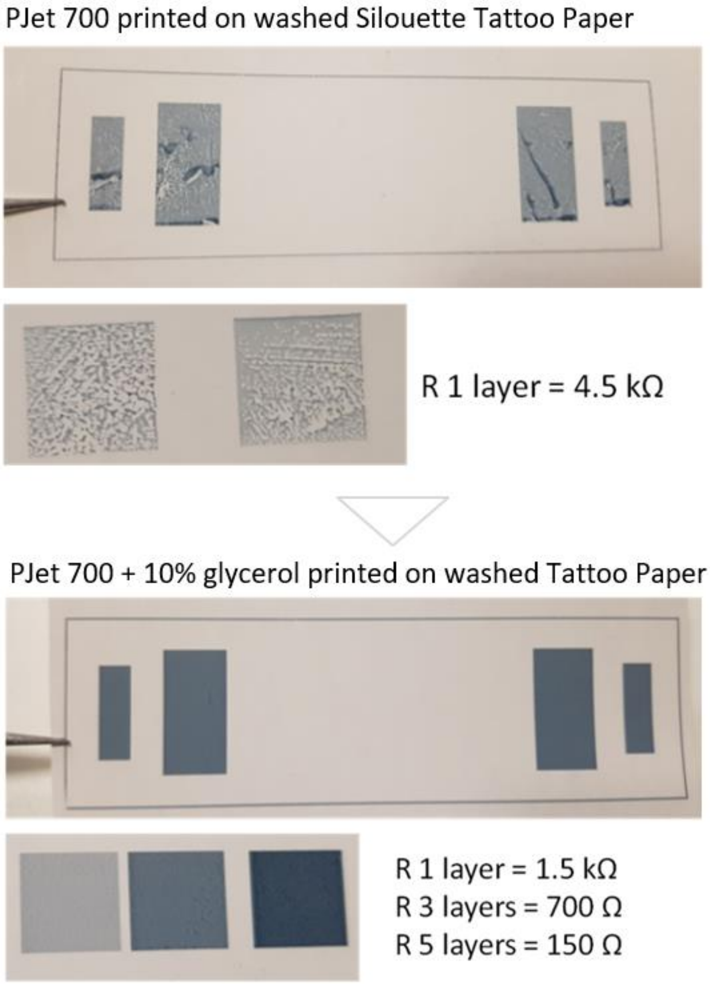

Figure 3. Example of samples obtained by printing PEDOT:PSS Clevios P Jet 700 as received on washed Silouette tattoo paper (top), and samples obtained by printing PEDOT:PSS Clevios P Jet with addition of 10%$_{vol}$ of glycerol (bottom). Typical value for electrodes' surface resistivity are reported.

Tattoo electrodes - material's properties

	PEDOT:PSS Layer	Tattoo Sub-strate	Silver Paste	Kapton
Bulk Modulus E [MPa]	1-2 10^3 [a]	42 [a]	≈ 100-200 [b]	2-2.7 10^3 [a]
Max Strength S_{max} [MPa]	---	---	≈ 10-26 [b]	≈ 100 [a]
Poisson's ratio ν	0.3 [c]	0.5 [c]	0.5 [c]	0.34 [a]
Max strain ε_{max}	5-10% (uniax) [b]	>10% [b]	>10% [b]	<1% [a]
Thickness [um]	0.4-0.6 [b]	1.5 [b]	15 [d]	25 [a]
Flexural rigidity D [N m $x10^{-9}$] [e]	0.0223	0.0236	56.3	4650

Table SI.1. Summary of typical mechanical properties for used materials.

[a] From literature (PEDOTPSS data from F. Greco et al. Soft Matter, 7: 10642, 2011) or technical datasheets

[b] Experimentally measured/verified

[c] Typical value for rubber-like (incompressible) materials is 0.5, typical value for rigid polymers is 0.3

[d] Nominal value for screen printing from silver paste material datasheet

[e] Calculated.

Transthoracic impedance measure and thermistor control – Details

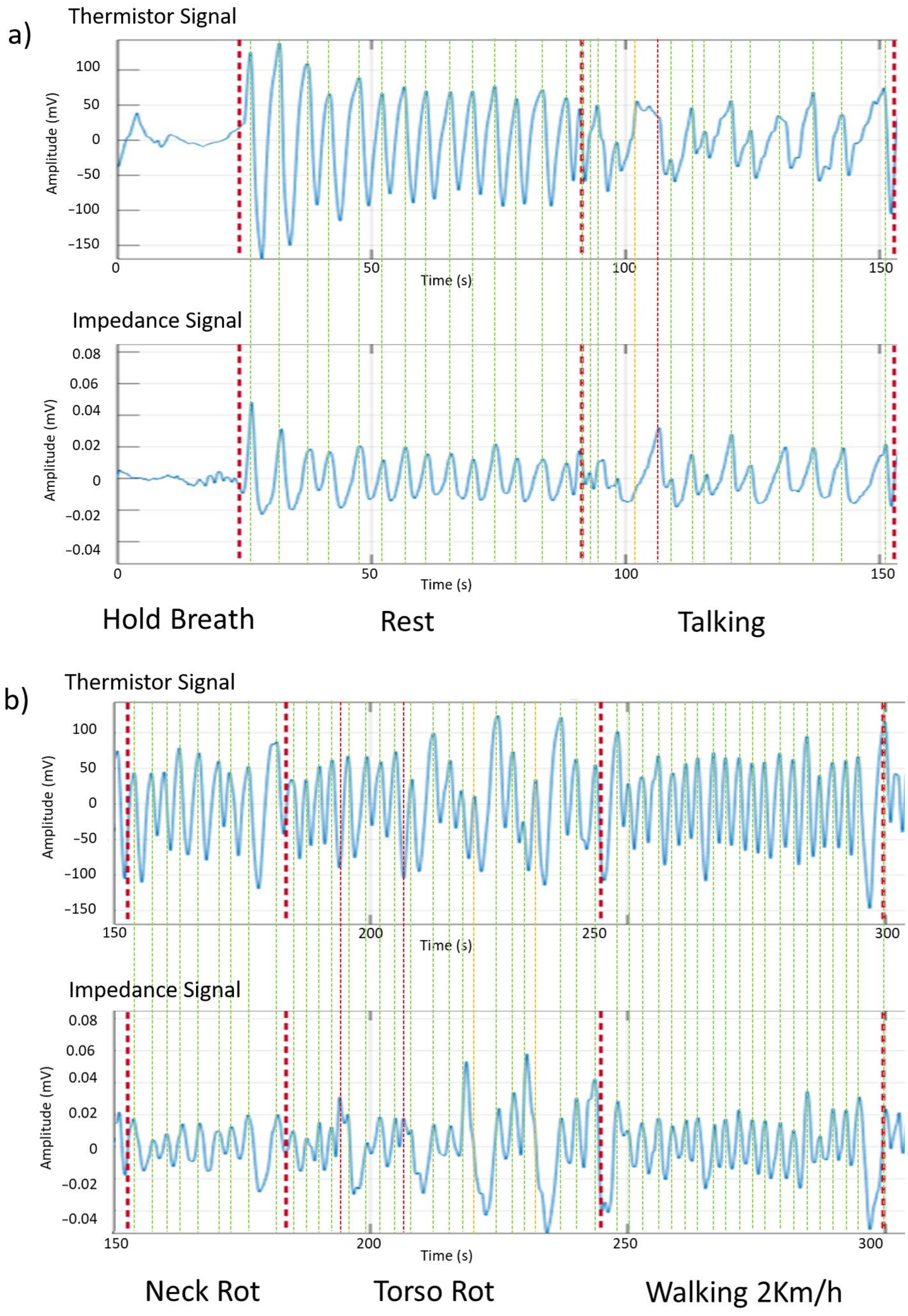

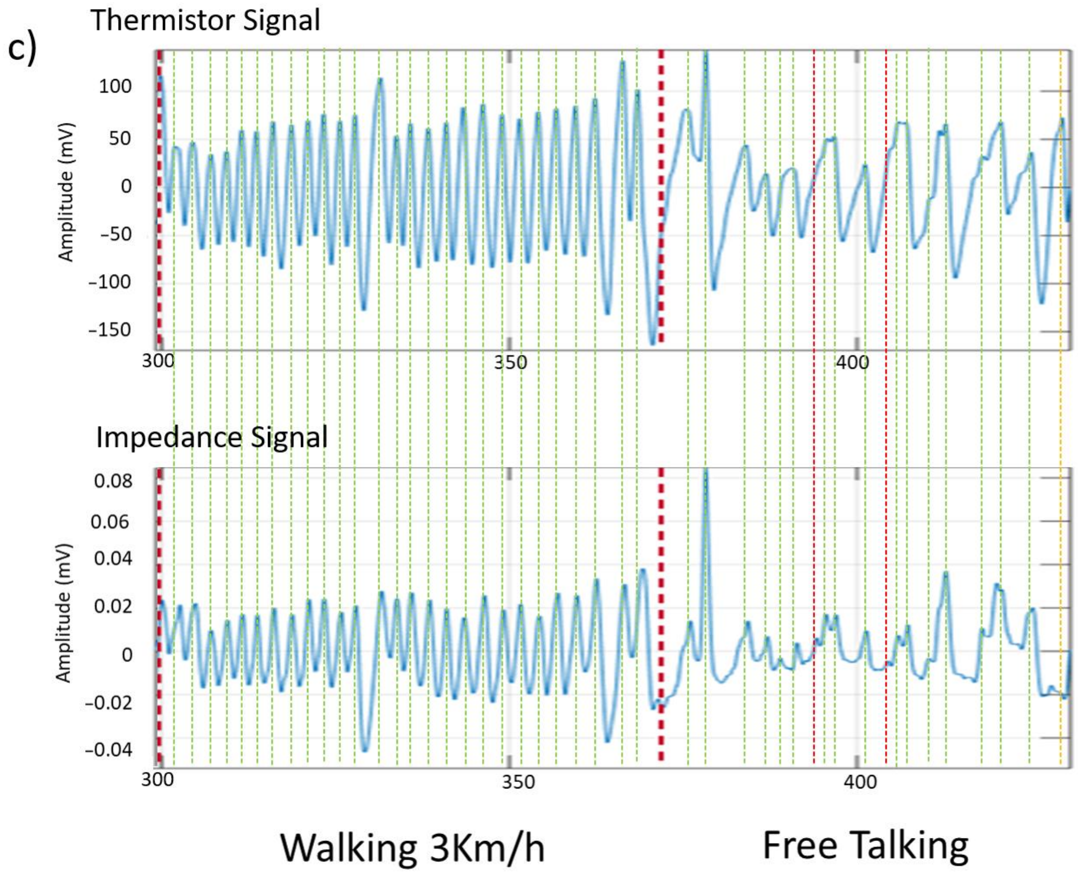

Figure 4. a-c) Details of transthoracic impedance measurement (and thermistor control) acquired while performing defined routines as presented in Figure 3 of the main text, showing the correspondences of respiratory acts detection (peaks of the curves). Green dashed lines show correct correspondence, orange dashed lines show false negative (thermistor peak detection, no impedance peak detection), red dashed lines show false positive (impedance peak detection, no thermistor peak detection). Overall the detection accuracy is around 92%.

Stand-alone devices for Tattoo Electrode interface – Fabrication and details

Stand-alone device is built in a modular way to easily adapt to different types of measurement, having a BT microcontroller board based on AZ-BLE PSoC module (by Cypress) for data collection/elaboration and transmission connected to specific sensor boards for analog front-end for each different application. Two different devices have been designed, one specific EMG measurements and another one specific for ECG measurement. Both devices have the same BT microcontroller HW module (but with different control firmware). Both device have the same housing and assembling procedures (see Figure SI5), and can be connected with a PC via Bluetooth through a suitable receiving dongle, where transmitted data is visualised in real time by means of a custom software interface.

Three electronic board have been developed for assembling the two devices: one Bluetooth Control Board (common to the two devices, see details in Figure SI6), one EMG Sensor Board (for EMG device, see details in Figure SI7) and one ECG Sensor Board (for ECG device, see details in Figure SI8). Some specific detail on the electronic of analog front-end architecture for both EMG and ECG device is provided in the follow.

EMG measurements device – Analog front-end

The analog front-end (Figure SI7) used for signal conditioning is composed by a first differential amplification stage with a gain G1 = 10, followed by a rectification stage, an active filter module and finally by a second amplification stage with tuneable gain. The output signal is acquired by BT microcontroller board with 12 bit of resolution (full span 3V), further elaborated (the derivative signal is calculated at 50 Hz update rate) and transmitted via BT with a UART (Universal asynchronous receiver-transmitter) over BT protocol to a PC where it is visualised in real time by means of a custom software interface

ECG measurements device – Analog front-end

The analog module for ECG detection (Figure SI8) is different from the previous one presented for ECG (while the BT microcontroller board is the same, and it is specifically built around the MAX30003 chip (Maxim Integrated, U.S.), a monolithic biopotential, analog front-end for clinical and fitness applications, providing ECG waveforms and heart rate detection. The MAX30003 is connected with the microcontroller through a high speed digital SPI interface, guaranteeing up to 500 sps (128 sps in our specific implementation) of waveform sampling with a resolution up to15 bit and 5μV$_{P-P}$ noise.

Stand-alone devices' fabrication – HW, FW, SW open source files and assembling process

All the material needed to build both stand-alone devices is made available as open source at the following repository: https://doi.org/10.5281/zenodo.4382056

The schematics and layouts files, developed with Eagle 6.3, are freely available at the repository (*"ElectronicBoards.zip"*), with complete bill of components.

The casing, common to the two device, has been 3D printed by stereo-lithography. STL files of device's external case (composed by two pieces, see Figure SI5) can be found at the repository (*"Device_External_Case STL.zip"*) with additional details for assembling (*"Assembly_Details.pdf"*).

Once fabricate the printed circuit boards and the external case, the assembly was performed as follow: - four magnets are inserted and glued in the holes if the bottom part of the case;

- two SMD dual-in-line pin connectors (slightly trimmed to fit in case) are glued on the bottom case;
- with conductive ink/paste are connected the pins with the magnets, to guarantee the electric connection mediated by magnetic interlocking;
- the sensor board (EMG or ECG) is inserted in the pins, and soldered to them
- the control board is staked on the Sensor board, including in the middle a small 50 mAh LiPo battery
- the battery is soldered to the control board
- the top part of the case is mounted and the device is ready for operations.

Each device is programmed with a specific firmware. Also the USB-BT dongle for PC Bluetooth connection (CY5677 CySmart BLE USB Dongle, by Cypress) need a specific firmware reprogramming. Devices' firmware and dongle's firmware are all developed with PSoC Creator 4.2 (Cypress), and are freely available at the repository (*"EMG_BLE_Device.zip"*, *"EGG_BLE_Device.zip"*, *"Dongle_BLEcentral.zip"*).

Once programmed the devices can be connected with the PC for data transmission and acquisition. The graphic user interface software, developed in Visual Basic .NET 2017 (Microsoft), is freely available at the repository (*"TestChart_ECG+EMG.zip"*).

Finally, DXF files for ECG and EMG tattoo printing/cutting can be found at the repository (*"TattooLayout.zip"*). Assembly of the tattoo electrodes can be done by following the process reported in Figure SI2.

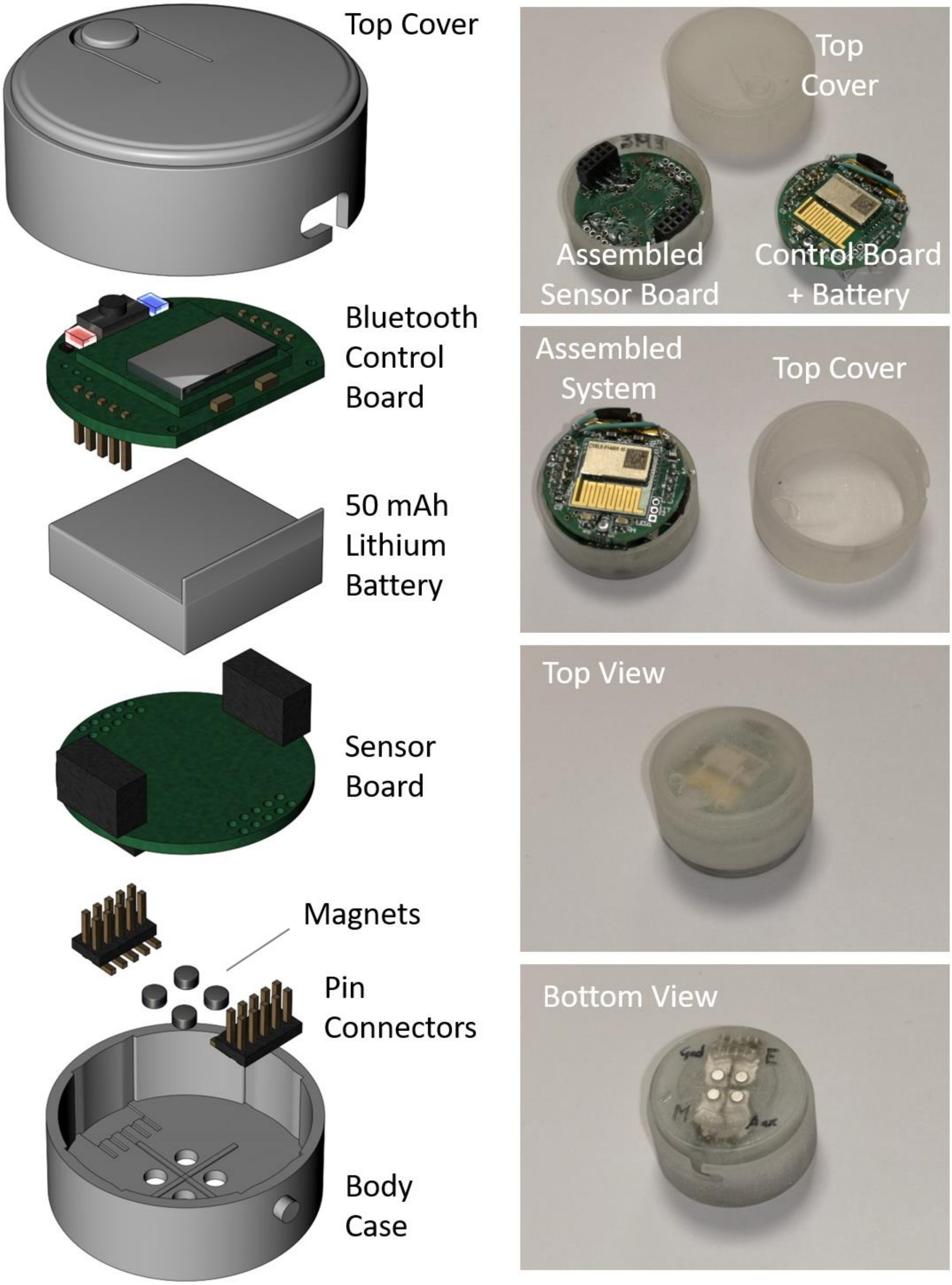

Figure 5. EMG /ECG BT device overview and pictures at different stage of assembly.

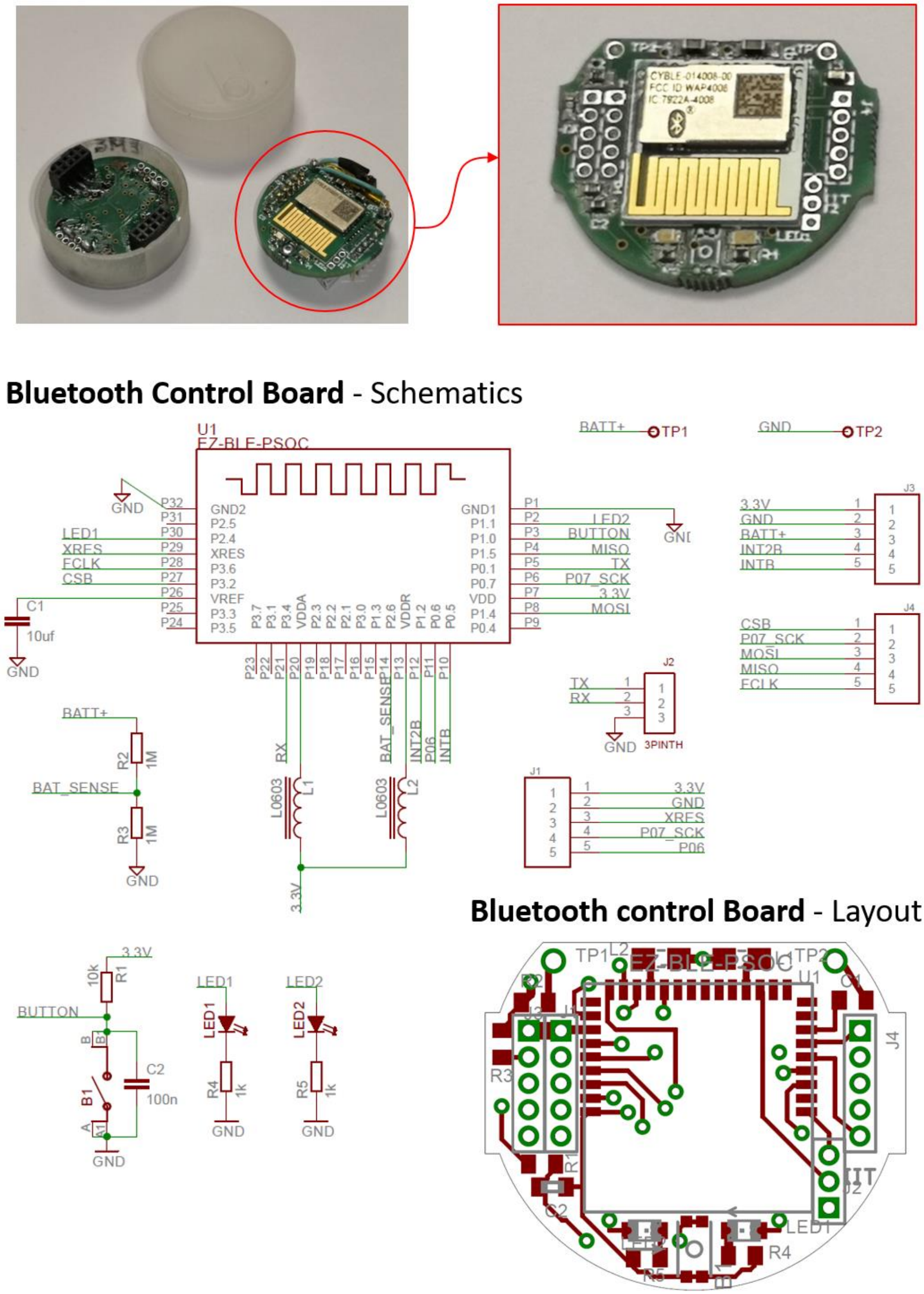

Figure 6. Bluetooth control board schematics and layout.

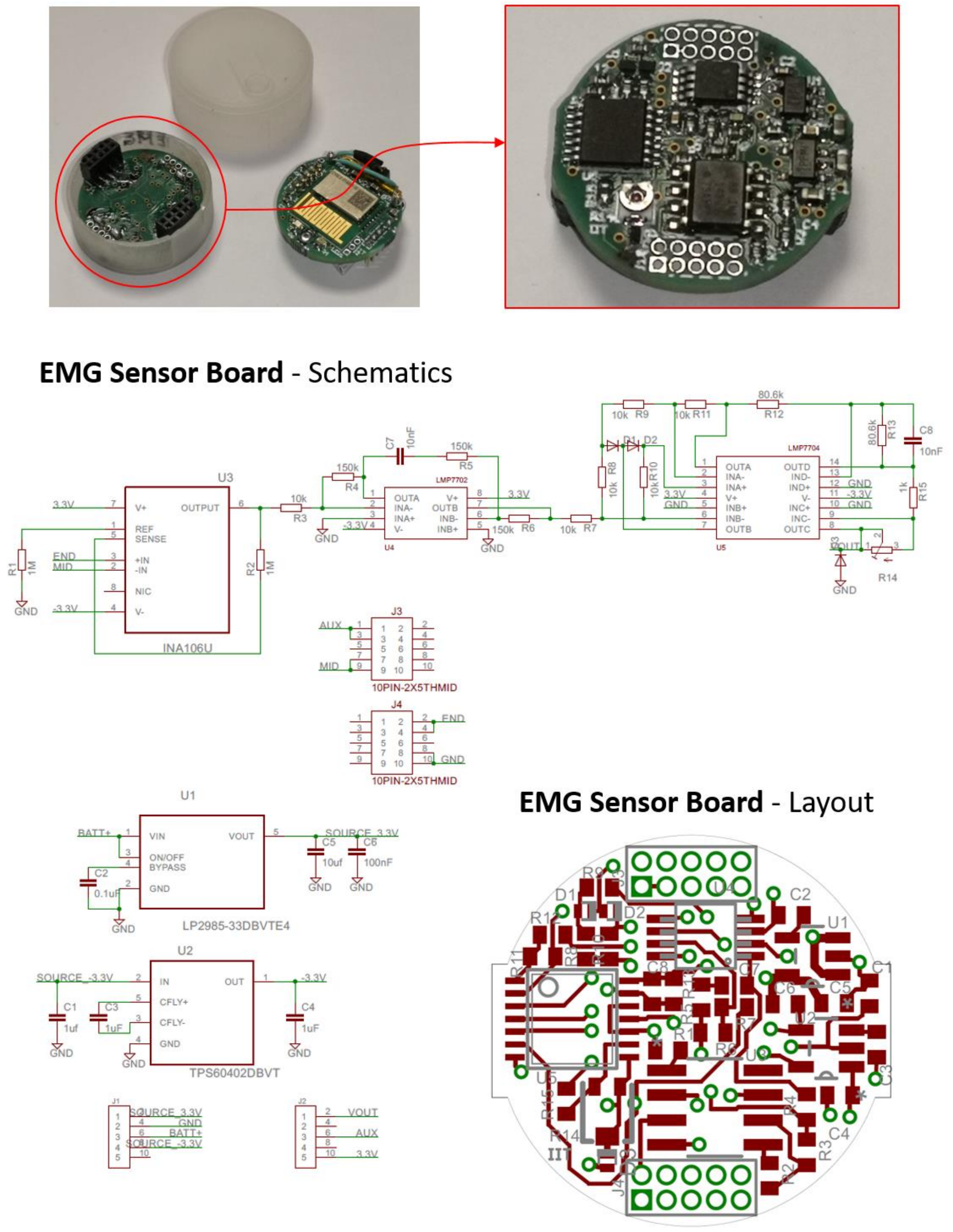

Figure 7. EMG sensor board schematics and layout - Signal conditioning schematic was partially based on the schematics available in https://www.instructables.com/id/Muscle-EMG-Sensor-for-a-Microcontroller/.

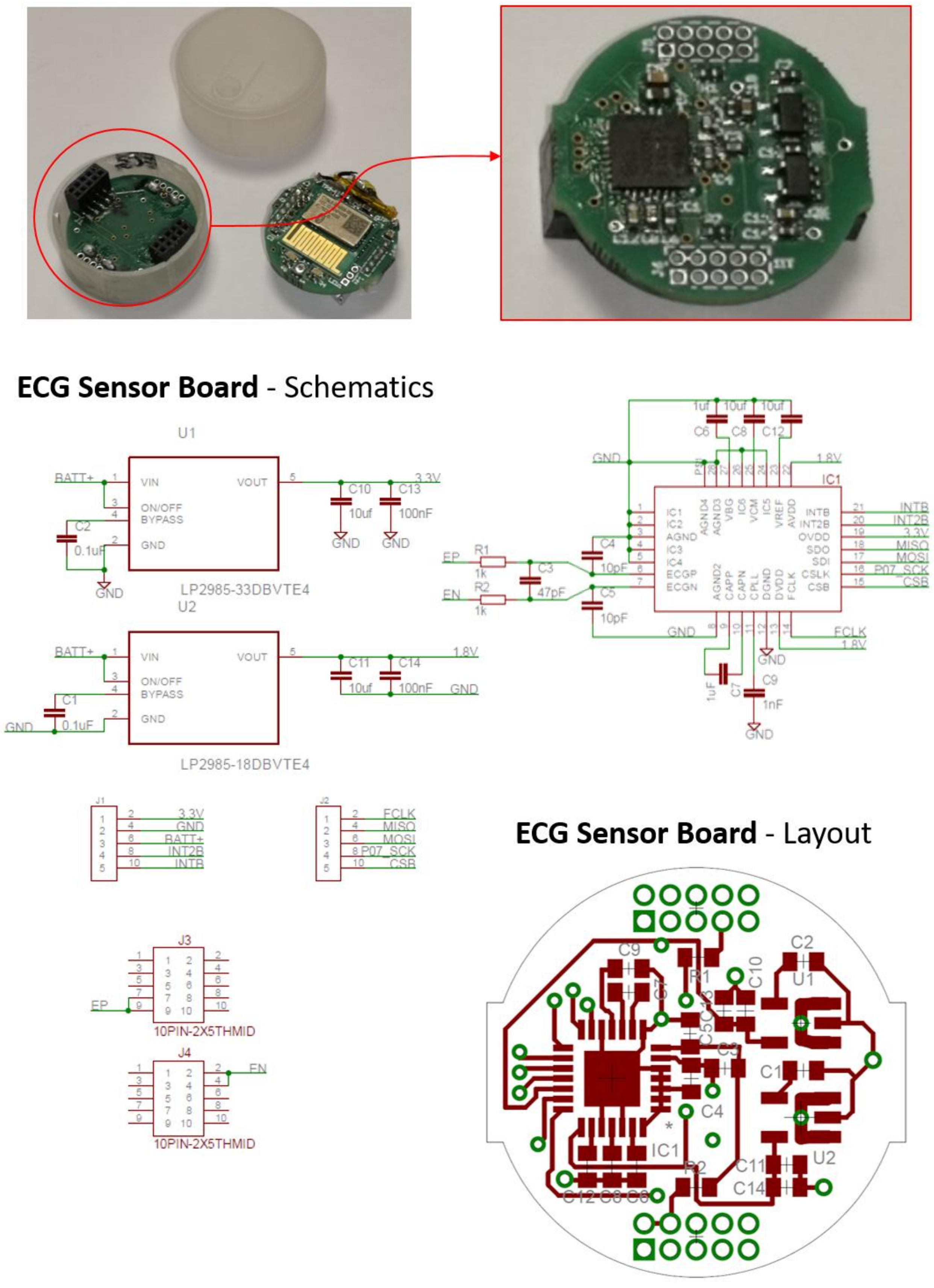

Figure 8. ECG sensor board schematics and layout – ECG signal conditioning is based on MAX30003 analog frontend.

ECG Comparison - Details

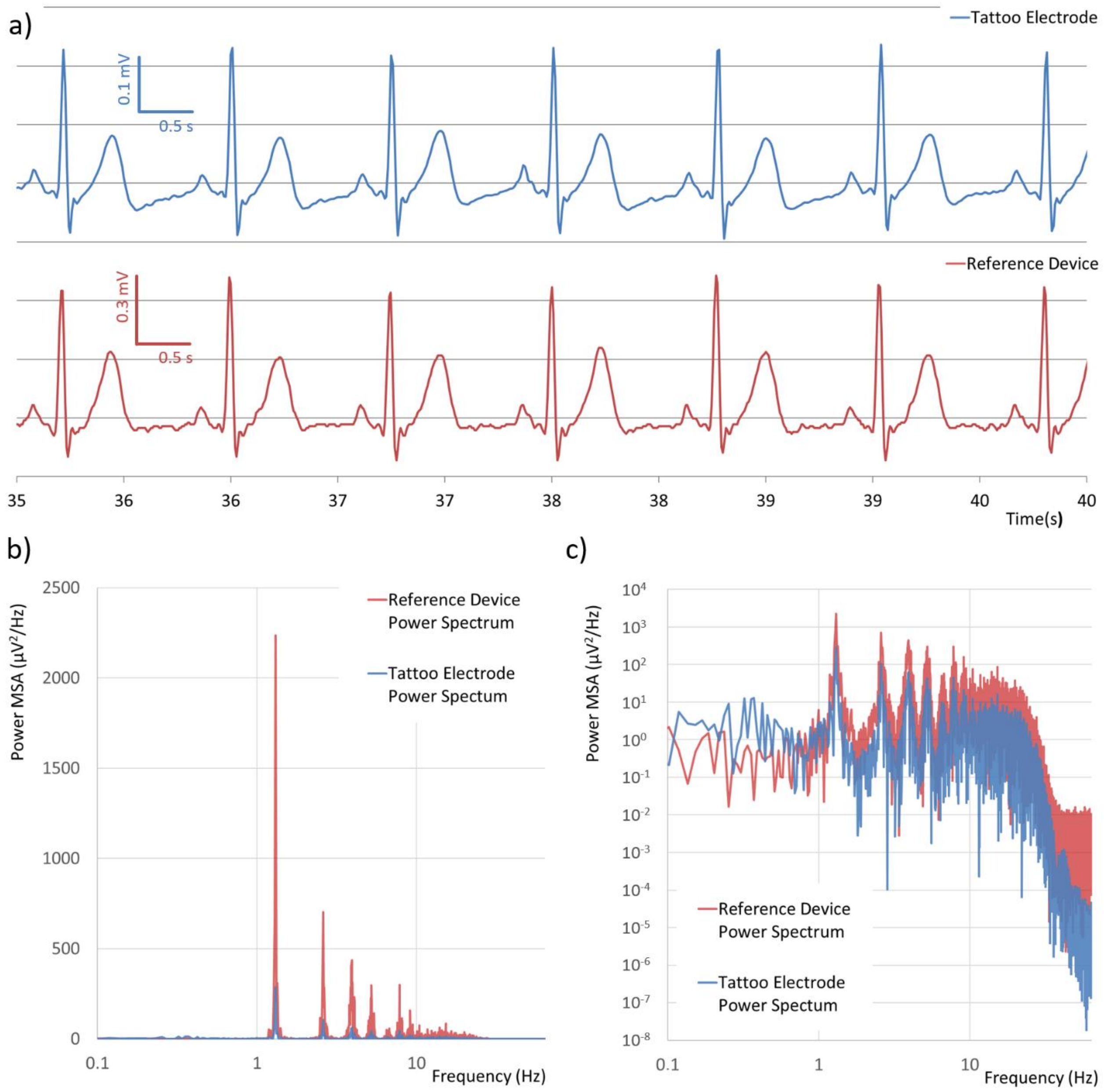

Figure 9. a) Detail of ECG acquisition from tattoo electrodes by BLE device and reference device with standard electrodes; (b) Power spectrum at mean squared amplitude(MSA) of the ECG signal calculated on 60 seconds sample, both for tattoo electrode + BLE device and reference device; (c) same spectrum with different logarithmic scaling, for clarity of comparison.

Supporting Information Video

List of videos:

1. SV#1 Video of tattoo stretching test while shaking
2. SV#2 Tattoo electrodes release on skin with sponge and EMG signal acquisition
3. SV#3 EMG demo with shaking
4. SV#4 EMG RC car control demo
5. SV#5 ECG comparative demo

High resolution SI Video also available for download from here: https://gitlab.iit.it/Virgilio.Mattoli/tattoo-electrode-device/-/tree/master/SI%20Videos
